# Mitochondria-Targeted and Resveratrol-Loaded Dual-Function Titanium Disulfide Nanosheets for Photothermal-Triggered Tumor Chemotherapy

**DOI:** 10.1186/s11671-019-3044-5

**Published:** 2019-06-21

**Authors:** Sen Xiang, Kaifang Zhang, Guanghua Yang, Dongdong Gao, Chen Zeng, Miao He

**Affiliations:** The First Department of Oncology, Zhumadian Central Hospital, 747 Zhumadian Zhonghua Road, Zhumadian, 463000 China

**Keywords:** Titanium disulfide, IR780 iodide, Mitochondria target, Apoptosis, NIR-triggered chemotherapy

## Abstract

A subcellular organelle-targeted delivery of anti-cancer drugs is a promising strategy to maximize the anti-cancer effects and minimize the adverse effects. Herein, we prepared a mitochondria-targeted drug delivery nanoplatform based on IR780 iodide (IR780) and titanium disulfide (TiS_2_) nanosheets. Due to the large specific surface area of TiS_2_ nanosheets, the nanoplatform could highly load anti-cancer drug resveratrol (RV). The as-prepared nanocomposite (IR780-TiS_2_/RV) was used for an efficacious photothermal-triggered tumor chemotherapy. IR780-TiS_2_/RV showed satisfactory stability and biocompatibility, and the loading ratio of RV and IR780 was about 112% and 56%, respectively. Upon the near-infrared (NIR) irradiation, the heat generated by IR780-TiS_2_/RV could trigger the RV release. Due to the conjugation with the mitochondria-specific IR780, IR780-TiS_2_/RV could target and accumulate in mitochondria and release RV when triggered by NIR to decrease the mitochondrial membrane potential, rapidly induce the upregulation of key intrinsic apoptotic factors such as cytochrome c, and initiate the caspase cascade, thereby achieving the chemotherapeutic effect. The IR780-TiS_2_/RV nanocomposite was demonstrated to have a high anti-tumor efficacy in vitro and in vivo as well as no remarkable tissue toxicity. We believe our study demonstrates that the NIR-triggered IR780-TiS_2_/RV nanoplatform could be a promising chemotherapeutic agent in clinical practice.

**Electronic supplementary material:**

The online version of this article (10.1186/s11671-019-3044-5) contains supplementary material, which is available to authorized users.

## Introduction

Cancer remains a life-threatening disease and accounts for high mortality rates worldwide [1]. Surgery, chemotherapy, radiation, and hormonal therapy are still the main therapeutic methods used in clinical practice [2, 3]. Of these methods, chemotherapy is a widely accepted treatment option by clinicians and patients alike because of its relatively high efficacy [4, 5]. Chemotherapy is associated with some serious problems, for example, drug resistance, tumor recurrence, and non-specificity [5–7]. Therefore, developing new chemotherapeutic agents and strategies to overcome these obstacles is of great urgency [8]. In recent years, loading an anti-cancer drug on a functionalized carrier that can simultaneously achieve a targeted delivery and a controlled release of the drug has become a popular approach to maximize the therapeutic effect and decrease the side effects [9–11]. A large specific area that can provide a better drug loading ratio is essential for an excellent drug carrier [12].

In addition, in order to improve the specificity of the drug, the carrier is usually modified with a cell-targeting molecule, such as cell surface receptor-targeting folic acid and glutathione [13–17]. Since many chemotherapeutic drugs act on specific subcellular organelles, designing an organelle-specific delivery system could remarkably augment the therapeutic effect and reduce the adverse effects [18–20]. Consequently, the selection of the targeted location inside the tumor cells is vital for the drug delivery system. Mitochondria are not only an “energy factory” of cells but also a key target of chemotherapeutic drugs which target the mitochondria to initiate the intrinsic apoptotic pathway [21–23]. Hence, developing a mitochondria-targeting anti-cancer drug delivery system could be vital for more effective cancer chemotherapy. Up to now, various kinds of drug delivery systems have been designed, such as mesoporous silica, carbon-based materials, and protein [17, 24–26]. Although these carriers have been reported to be efficient for drug delivery and tumor therapy, more new high-efficient drug delivery systems are still highly desired.

**Scheme 1:**
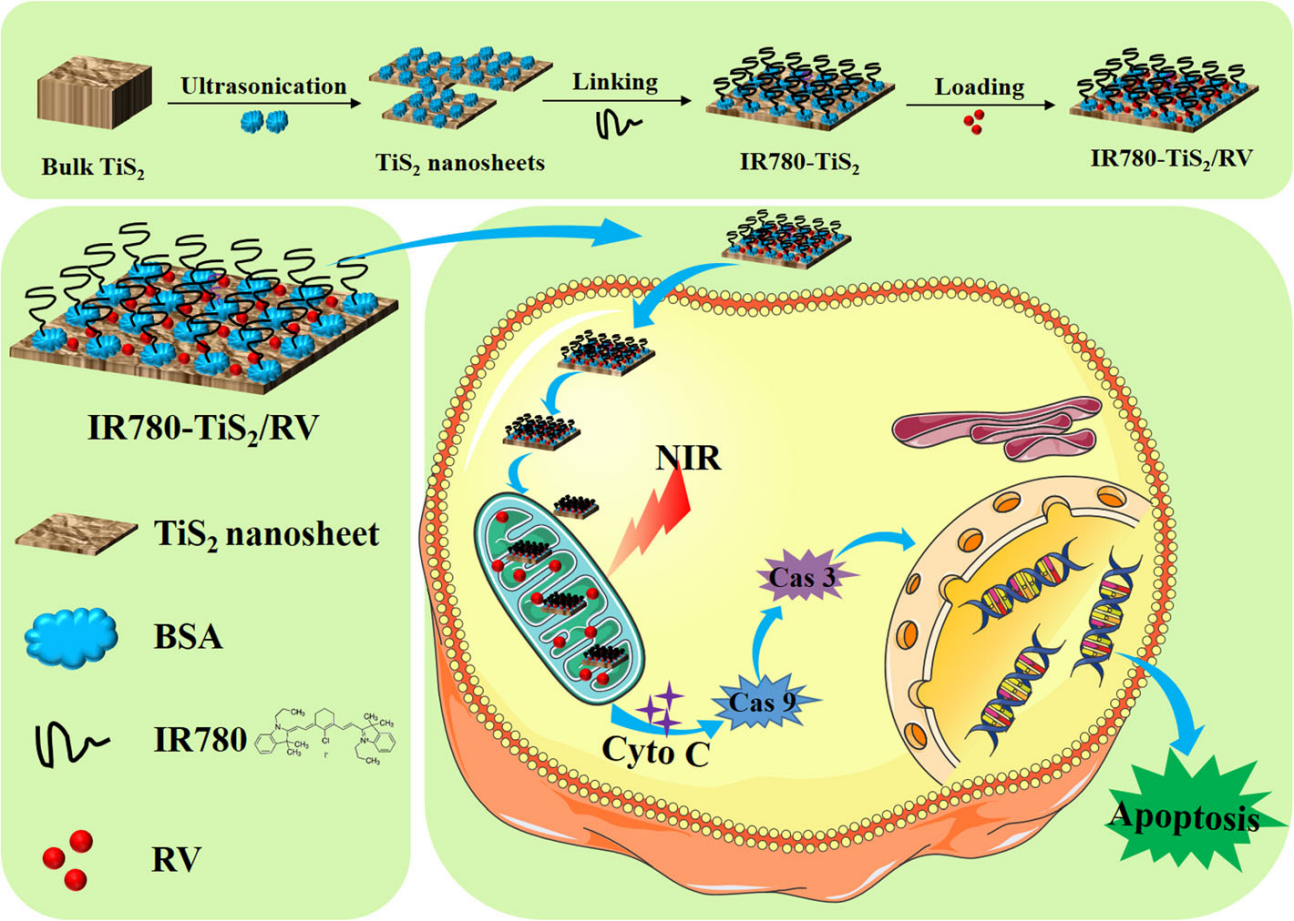
The preparation schematic of IR780-TiS_2_/RV, which was used as a NIR-triggered drug delivery system for tumor chemotherapy mediated by intrinsic apoptosis pathway

In this study, we constructed a mitochondria-targeting and NIR-triggered drug delivery nanoplatform based on two-dimensional titanium disulfide (TiS_2_) nanosheets for controlled and targeted tumor chemotherapy. TiS_2_ is a member of transition metal dichalcogenides, which have a large specific area [27–29]. After exfoliation by protein-assisted ultrasonication, the surface of TiS_2_ nanosheets is modifiable by other functional molecules via covalent or non-covalent interactions. In addition, the tumor mitochondria-specific molecule IR780 (IR-780 iodide) was chosen to modify the TiS_2_ nanosheets. IR780 endows the nanosheets with the mitochondria-targeting capability, which is through both the organic-anion transporting polypeptide-mediated active transport and the lipophilic cation feature [30, 31]. It was reported that IR780 as a lipophilic cation, showed more accumulation in the mitochondria of tumor cells owing to the high magnitude of mitochondrial membrane potential in tumor cells than normal cells [32, 33]. In addition, IR780 is also a near-infrared responsive photothermal agent. The as-prepared nanoplatform, termed IR780-TiS_2_, was confirmed to be biocompatible and could effectively load the anti-cancer drug resveratrol (RV) (IR780-TiS_2_/RV) [24]. The IR780-TiS_2_/RV possessed a mitochondria-targeting capability and a photothermal effect. NIR was applied as an external stimulus to trigger the local drug release upon NIR irradiation. In vitro and in vivo experiments showed that IR780-TiS_2_/RV has a highly efficient chemotherapy effect. Further study of the mechanism revealed that the cell death induced by IR780-TiS_2_/RV was via the mitochondria-mediated intrinsic apoptosis pathway. This mitochondria-targeted drug delivery system (Scheme 1) could be a potential chemotherapeutic agent in future clinical application.

## Materials and Methods

### Materials

Sigma-Aldrich provided the IR-780 iodide (IR780), TiS_2_ powder, fluorescein isothiocyanate (FITC) (St. Louis, MO, USA). Bovine serum albumin (BSA ≥ 98.0%) was purchased from BioSharp Co., Ltd. (Korea). Cell Counting Kit-8 (CCK-8) was procured from Dojin Chemical Laboratory Co., Ltd. (Kumamoto, Japan). Cell culture reagents including DMEM medium and fetal bovine serum (FBS) were provided by Gibco (Invitrogen, Carlsbad, CA, USA). Chemicals were obtained from Sigma-Aldrich (Shanghai, China).

### Synthesis of IR780-TiS_2_/RV

The first step was the preparation of water-soluble TiS_2_ nanosheets according to previous report [34]. In detail, 5 mg bulk TiS_2_ was mixed with 5 mL water and stirred for 20 min. The TiS_2_ water suspension was then added into 5 mg BSA and was processed under ice-bath ultrasonic dissociated using a 500 W and 20 kHz tip sonication (Sonics, VCX130, USA) for 6 h. Afterwards, the prepared mixture was centrifuged at 12,000 rpm for 10 min, resulting in TiS_2_ nanosheets in the supernatant.

Second, TiS_2_ nanosheets were conjugated with IR780. Triethylamine (TEA) was applied as an acid-binding agent. Five milligrams of IR780 was dissolved in 2 mL DMSO and stirred at 60 °C for 8 h with the addition of TEA. The mixture suspension was dialyzed in distilled water for 2 days and then was centrifuged at 9000 rpm for 8 min to remove the aggregated product. The supernatant was collected, resulting in IR780-TiS_2_.

At last, RV was loaded onto IR780-TiS_2_. IR780-TiS_2_ (1 mg/ml) was mixed with RV (2 mg/ml, dissolved in DMSO), which was slightly stirred overnight at room temperature. Afterwards, DMSO and free RV were eliminated via dialysis in distilled water overnight to give IR780-TiS_2_/RV. Based on literature, the RV loading ratio detected by an UV-vis spectrophotometer (UV-2550, Shimadzu, Japan) and calculated according to the following equation:

$$ \mathrm{RV}\ \mathrm{loading}\ \mathrm{ratio}\ \left(\%\right)=\frac{A_a-{A}_b}{A_c}\times 100\% $$

where *A*_*a*_ (mg), *A*_*b*_ (mg), and *A*_*c*_ (mg) represent the initial, unbound RV, and the IR780-TiS_2_, respectively.

Bruker TENSOR 27 Fourier transform infrared spectroscopy (FTIR) spectrometer (Bruker Optics Ltd., Coventry, UK) was used to detect the chemical structure of TiS_2_, IR780-TiS_2_, and R780-TiS_2_/RV. Brunauer-Emmett-Teller (BET) analysis was carried out to determine the specific surface area of the samples, calculated from N_2_ adsorption result via a surface area analyzer (Quantachrome ChemBET-3000) based on the BET equation.

### Cell Line and Cell Culture

The mouse colon cancer cells CT26 were obtained from the Chinese Academy of Sciences Cell Bank of Type Culture Collection (Shanghai, China). The cells were cultured in complete DMEM media (10% FBS + 90% DMEM) in a humidified incubator with 5% CO_2_ at 37 °C.

### In Vitro Localization of IR780-TiS_2_/RV

FITC was used to label IR780-TiS_2_/RV or TiS_2_/RV. In brief, FITC was dissolved in ethanol solution (2.0 mg/mL) and mixed with IR780-TiS_2_/RV or TiS_2_/RV aqueous solution (1.0 mg/mL) under a 4-h stir in dark environment at room temperature. The mixture was dialyzed in distilled water overnight to remove the redundant FITC and ethanol, resulting in FITC-labeled IR780-TiS_2_/RV or TiS_2_/RV solution. To confirm the mitochondrial co-localization of nanoparticles in vitro, the cells treated with FITC-labeled IR780-TiS_2_/RV or TiS_2_/RV for 5 h and stained by mitochondria-specific dye MitoTracker. Afterwards, the cellular internalization of IR780-TiS_2_/RV or TiS_2_/RV was observed using a CLSM (Ix81-FV1000, Olympus, Co.). In brief, CT26 cells were incubated with FITC-labeled TiS_2_/RV and IR780-TiS_2_/RV (with the same concentration of FITC) for 5 h. And then, the cells were treated with MitoTracker Red FM solutions (100 nM) at 37 °C for 30 min. After washing by PBS for three times, the cells were observed by a CLSM. ImageJ software was used to analyze the fluorescence intensity of cells.

### In Vitro NIR-Triggered Tumor Chemotherapy and Apoptosis Study

4 × 10^3^ cells/well CT26 cells in 96-well culture plates were treated with free RV, IR780-TiS_2_, IR780-TiS_2_/RV, and TiS_2_/RV with different RV concentrations for 5 h and then were irradiated with or without NIR for 3 min (808 nm, 0.3 W/cm^2^). After further 24-h incubation, the viability of treated cells was analyzed by CCK-8 assay. The treated cells were also stained by Rhodamine123 (Sigma) and analyzed by FCM (FC 500 MCL; Beckman Coulter, USA). Cell apoptosis detection was also performed by FCM analysis using Annexin V-FITC/PI apoptosis detection kit (Bender MedSystems, Vienna, Austria) as previously described.

### Western Blot

CT26 cells were treated with PBS (control), RV, TiS_2_/RV, IR780-TiS_2_/RV, and IR780-TiS_2_/RV + NIR (equivalent RV, 30 μg/mL; equivalent IR780, 0.5 μg/mL) for a 5-h incubation. After further a 24-h incubation, the cells were collected respectively of Western blot, which was based on the protocol reported previously [23]. Briefly, the cells were lysed and inhibited by a protease and Triton X-100. Sodium dodecyl sulfate-polyacrylamide gel electrophoresis was used to recover and separate proteins which were then shifted to a PVDF membrane and blocked using 5% fat-free milk. Afterwards, the diluted primary antibodies were incubated at 4 °C for 12 h, including cytochrome c (1/1000, Boster, Wuhan, China), cleaved caspase-3 (1/1000, CST), cleaved caspase-9 (1/1000, CST), and actin (1/1000, Mouse, Boster, Wuhan, China) and then incubated with a secondary antibody. Finally, ECL reagent was used to detect the proteins.

### Animal Model

To establish CT26 subcutaneous tumor model, 1 × 10^7^ CT26 cells (100 μL, in PBS) were subcutaneously injected into the back of Balb/c nude mice. Tumor volume = length × width^2^/2. All animal procedures were performed in accordance with the Guidelines for Care and Use of Laboratory Animals of the National Institutes of Health and approved by the Animal Ethics Committee of Zhumadian central hospital (Henan, China).

### In Vivo Biodistribution

Biodistribution of IR780-TiS_2_/RV in the tumor-bearing nude mice was detected at 24 h post tail intravenous injection with IR780-TiS_2_/RV (150 μL, 6 mg/kg). The major organs including the heart, liver, spleen, lung, kidney, and tumor were weighed and digested by aqua regia solution. The Ti element content in these tissues was quantified by ICP-OES.

### In Vivo NIR-Triggered Tumor Chemotherapy

CT26 tumor-bearing mice were randomly separated into six groups (*n* = 6), including saline, saline +NIR, RV, IR780-TiS_2_/RV, TiS_2_/RV, and IR780-TiS_2_/RV + NIR (equivalent RV, 1 mg/kg; equivalent IR780, 0.5 mg/kg). The NIR irradiation condition is 808 nm, 0.3 W/cm^2^, and 3 min. During 30 days of treatment, tumor volumes and mice weights were recorded every 3 days. After the treatment, the organs including the heart, liver, spleen, lung, and kidney in various groups were fixed and stained by H&E.

### Statistical Analysis

The results were presented as the mean ± standard deviation. Student’s *t* test was used to examine significant differences between two groups. *P* < 0.05 was regarded as significant and *P* < 0.01 was considered very significant.

## Results and Discussion

### Preparation and Characterization of IR780-TiS_2_/RV

**Fig. 1:**
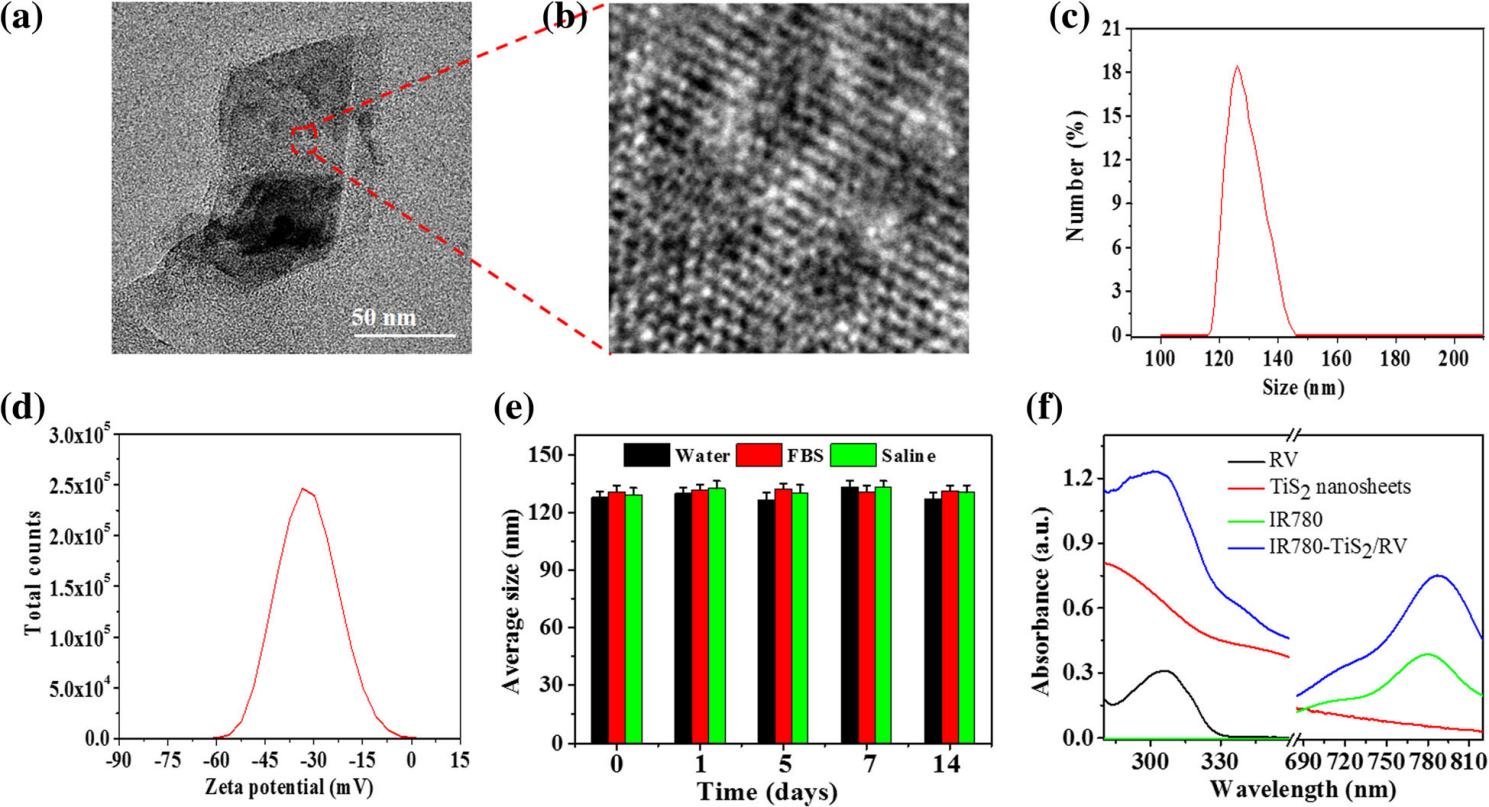
**a** The TEM image of IR780-TiS_2_/RV. **b** The high-resolution TEM image of IR780-TiS_2_/RV. **c** The size distribution and **d** zeta potential distribution of IR780-TiS_2_/RV. **e** The hydrodynamic particle size change of IR780-TiS_2_/RV in water, FBS, and saline over 2 weeks. **f** The absorption spectra of RV, IR780, TiS_2_ nanosheets, and IR780-TiS_2_/RV

To prepare IR780-TiS_2_/RV, firstly, biocompatible TiS_2_ nanosheets were prepared through a bovine serum albumin (BSA) and ultrasonication-assisted exfoliation method as reported previously [34, 35]. Next, IR780-TiS_2_ was synthesized in a substitution reaction between a chlorine atom of IR780 and amino groups of BSA absorbed on the TiS_2_ nanosheets using an acid-binding agent TEA. At last, the IR780-TiS_2_ nanoplatform loaded the anti-cancer drug RV via physical absorption. The schematic diagram of IR780-TiS_2_/RV is shown in Scheme 1. Additional file 1: Figure S1 shows the TEM image of the protein-assisted exfoliated TiS_2_, which was a displayed nanosheet structure. The XRD pattern of the TiS_2_ nanosheets were also detected, which indicated good crystallinity of the prepared TiS_2_ nanosheets, consistent with the observations from TEM (Additional file 1: Figure S2). After functionalization, the IR780-TiS_2_/RV nanocomposite had a flake-like morphology with a lattice spacing of 0.25 nm as shown by transmission electron microscopy (Figs. 1a, b) and an average diameter of about 123.6 nm and zeta potential of − 37.2 mV as detected by Nanosizer (Malvern Instruments) (Fig. 1c, d). After a 2-week storage in water, FBS, or saline, the average size of IR780-TiS_2_/RV remained constant at approximately 123 nm (Fig. 1e) indicating the stability of IR780-TiS_2_/RV, possibly due to adhesion of BSA on the surface of the TiS_2_ nanosheets. Figure 1f shows the absorbance spectra of free IR780, free RV, TiS_2_ nanosheets, and IR780-TiS_2_/RV. The absorption spectrum of IR780-TiS_2_/RV showed the peaks of free IR780 and free RV, indicating that both IR780 and RV were successfully conjugated with the TiS_2_ nanosheets. The RV loading ratio of IR780-TiS_2_ was about 112% (*W*/*W*), which was achieved through non-covalent interactions (e.g., *π*–*π* stacking and hydrophobic interactions). Furthermore, according to the BET result, the surface areas were calculated to be 15.2 m^2^/g and 122.1 m^2^/g for bulk TiS_2_ and TiS_2_ nanosheets, respectively. The exfoliated TiS_2_ nanosheets show a much higher BET surface area than that of the bulk TiS_2_, which provide large active area for drug loading. The loading was high likely due to the large specific surface area of and the BSA adhesion to the IR780-TiS_2_ nanosheets [23]. To further confirm RV and IR780 were loaded in TiS_2_ nanosheets, the FTIR spectra of TiS_2_ nanosheets, IR780-TiS_2_, and IR780-TiS_2_/RV were measured. In Additional file 1: Figure S6, all the characteristic peaks of TiS_2_ nanosheets appeared in FTIR spectra of IR780-TiS_2_/RV. Moreover, three new peaks appeared (~ 3191 cm^−1^, ~ 1510 cm^−1^, 1230 cm^−1^) in the spectrum of IR780-TiS_2_/RV, indicating that the existence of RV and IR780 [36, 37].

**Fig. 2:**
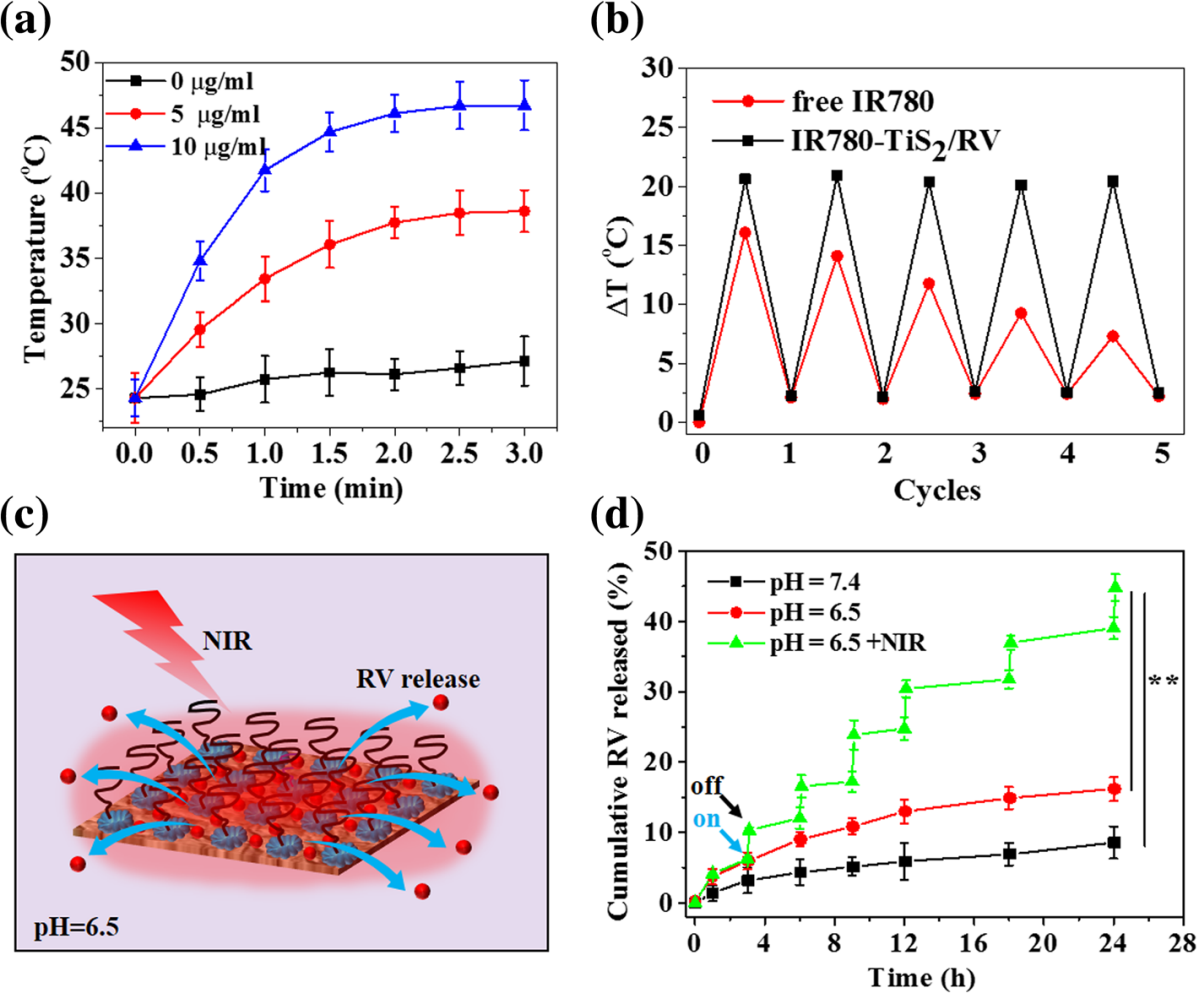
**a** The photothermal effect of IR780-TiS_2_/RV under the NIR irradiation (808 nm, 0.3 W/cm^2^) for 3 min. **b** Temperature change of IR780 and R780-TiS_2_/RV after five cycles of NIR irradiations (808 nm, 0.3 W/cm^2^, 3 min). **c** The schematic of photothermal-triggered RV release. **d** Release kinetics of RV from IR780-TiS_2_/RV in PBS buffer (pH = 7.4 and 6.5) with or without NIR irradiation (808 nm, 0.3 W/cm^2^). **P < 0.01, compared with pH = 7.4 and pH = 6.5 group

After a 3-min low-power NIR irradiation (808 nm, 0.3 W/cm^2^), the IR780-TiS_2_/RV solution temperature increased proportionately to the concentration of IR780-TiS_2_/RV (0, 5, 10 μg/ml), and the 10 μg/ml IR780-TiS_2_/RV solution reached the highest temperature of 47.6 °C (Fig. 2a). Additionally, IR780-TiS_2_/RV had retained its initial photothermal effect even after five cycles of NIR irradiation, while free IR780 showed a decreased photothermal effect (Fig. 2b). These results indicate the IR780-TiS_2_/RV nanocomposite has great photostability.

Next, the RV release ratio was measured under various conditions of pH and NIR irradiation (Fig. 2c, d). After 24 h, the RV releasing ratio was 8.56% under physiological conditions (pH 7.4), but it was significantly increased to 16.2% at pH 6.5, indicating that it can be enhanced by weakly acidic conditions. Furthermore, the RV release ratio was again significantly increased to 44.8% at pH 6.5 and a 3-min NIR irradiation (808 nm, 0.3 W/cm^2^). These results demonstrate that the NIR irradiation can be a controllable external stimulus to trigger the release of RV from IR780-TiS_2_/RV. This effect is likely due to the heat generated by IR780-TiS_2_ under NIR irradiation weakening the non-covalent adsorption interactions between RV and the IR780-TiS_2_ surface [35]. In addition, at acidic environment, the H+ could change the surface charge of TiS_2_ that alter the hydrophilic/hydrophobic balance of the nanoparticles [38, 39].

### In Vitro Localization of IR780-TiS_2_/RV

**Fig. 3:**
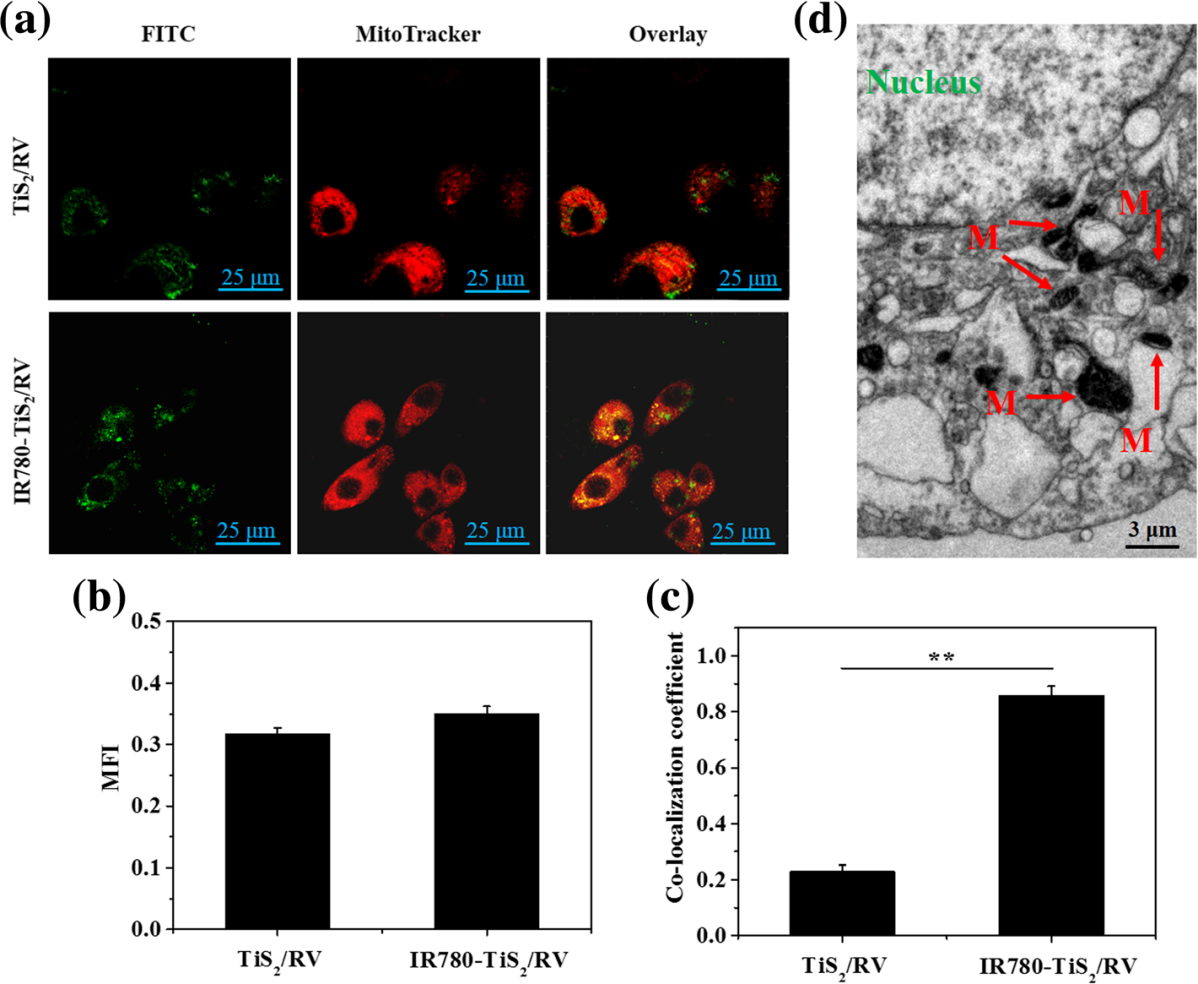
**a** The fluorescence images of CT26 cells incubated with FITC-labeled TiS_2_/RV or IR780-TiS_2_/RV for 5 h. Green fluorescence is the FITC signal, and red fluorescence is MitoTracker signal. **b** The corresponding mean fluorescence intensity (MFI) analysis of FITC-labeled TiS_2_/RV or IR780-TiS_2_/RV in cells in Fig. 3a. **c** The corresponding co-localization coefficient of FITC-labeled TiS_2_/RV or IR780-TiS_2_/RV and mitochondria in cells in Fig. 3a. M means mitochondria. **P < 0.01, compared with TiS_2_/RV group alone

To investigate the cell uptake and the intracellular distribution of IR780-TiS2/RV, the IR780-TiS_2_/RV nanoparticles were labeled with FITC, and a CLSM was used to visualize the intracellular fluorescence. After a 5-h incubation, TiS_2_/RV and IR780-TiS_2_/RV showed a similar fluorescence intensity in the cytoplasm (Fig. 3a, b), indicating that both nanocomposites could enter cells, likely via endocytosis. However, the IR780-TiS_2_/RV nanocomposite exhibited a greater intensity of yellow and green fluorescence when the FITC signal was merged with MitoTracker labeling (Fig. 3a, c). These results indicate that IR780-TiS_2_/RV can target mitochondria with a high efficiency. Additionally, the distribution of IR780-TiS_2_/RV within the cytoplasm was further confirmed using TEM, which showed retained nanoparticles in some mitochondria (Fig. 3d). Together, these results firmly confirm that IR780-TiS_2_/RV achieves good mitochondrial targeting, which further promotes mitochondrial drug accumulation, causing immediate cell toxicity. The mitochondria targeting was likely mediated by both the organic-anion transporting polypeptide-mediated active transport and the lipophilic cation feature, which made the nanoparticles highly accumulate in the mitochondria of tumor cells [30–33].

### In Vitro NIR-Triggered Tumor Chemotherapy

**Fig. 4:**
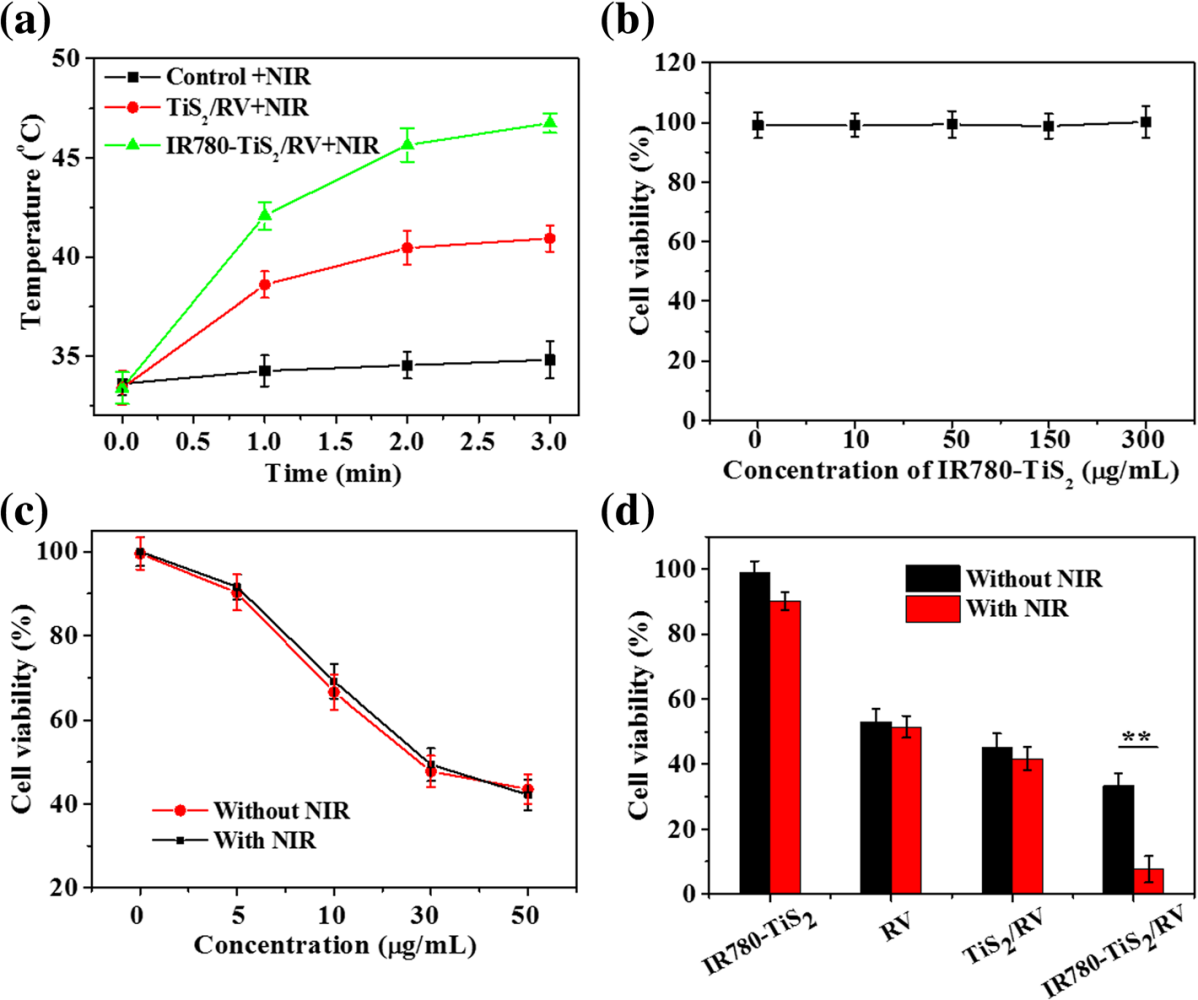
**a** The temperature change curves of PBS (control), TiS_2_/RV, or IR780-TiS_2_/RV-treated cells under a 3-min NIR irradiation (808 nm, 0.3 W/cm^2^). **b** In vitro cytotoxicity against CT26 cells treated with different concentrations of IR780-TiS_2_. **c** Cell viability of cells treated with RV with or without NIR irradiation (808 nm, 0.3 W/cm^2^) for 3 min. **d** Cell viability of cells treated with IR780-TiS_2_, RV, TiS_2_/RV, or IR780-TiS_2_/RV with or without NIR irradiation (808 nm, 0.3 W/cm^2^) for 3 min. **P < 0.01, compared with IR780-TiS_2_/RV without NIR group alone

First, the photothermal effect was evaluated in vitro. After a 5-h incubation, IR780-TiS_2_/RV showed a temperature increase of about 17 °C, while TiS_2_/RV displayed a temperature increase of about 10 °C (Fig. 4a). The photothermal effect was attributed to IR780 and TiS_2_ nanosheets. Demonstrating biocompatibility as a nanoplatform for drug loading, IR780-TiS_2_ displayed no pronounced cytotoxicity below the concentration of 300 μg/ml (Fig. 4b). Furthermore, RV with or without NIR irradiation had a similar concentration-dependent cell killing effect (Fig. 4c). At the same RV concentration, RV achieved the best cell killing effect when loaded by IR780-TiS_2_ and irradiated by NIR compared to all other conditions, i.e., free RV, free IR780-TiS_2_, and RV loaded by TiS_2_ (Fig. 4d). Interestingly, the unloaded IR780-TiS_2_ platform displayed no remarkable anti-cancer effect when exposed to NIR irradiation compared to that without NIR irradiation (Fig. 4d), indicating that the heat generated by IR780-TiS_2_ upon the NIR irradiation stimulus was mainly used to trigger the drug release which then killed the cells. These results suggest that IR780-TiS_2_/RV can release RV in the mitochondria when triggered by NIR irradiation and, thus, remarkably enhance the local concentration of RV within the mitochondria and achieve a greater tumor inhibiting effect.

### The Cell Death Mechanism

**Fig. 5:**
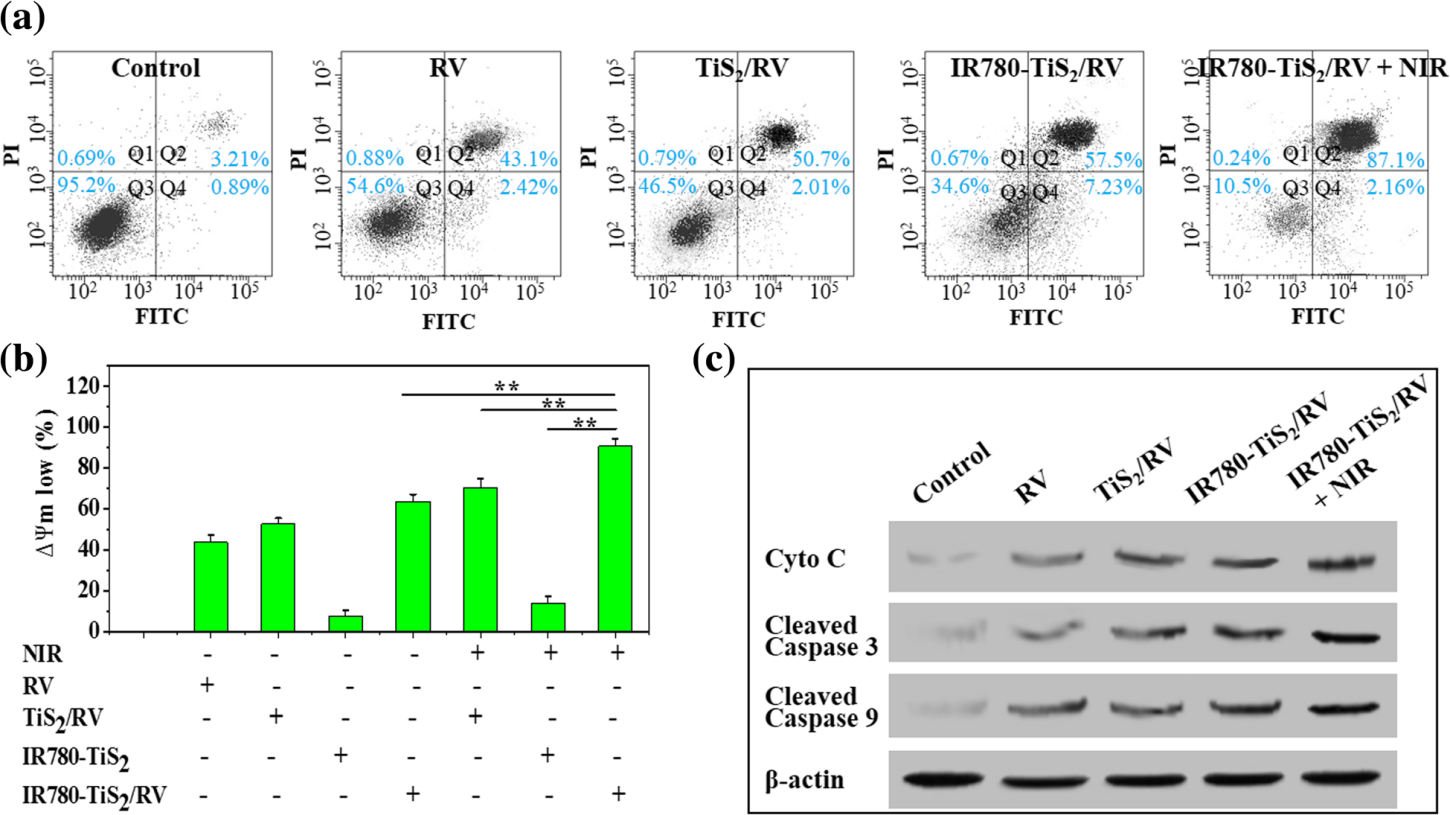
**a** Cell apoptosis of CT26 cells treated with PBS (control), RV, TiS_2_/RV, IR780-TiS_2_/RV, and IR780-TiS_2_/RV + NIR by FCM. **b** The change in mitochondrial membrane potential of CT26 cells treated with PBS (control), RV, TiS_2_/RV, IR780-TiS_2_/RV, and IR780-TiS_2_/RV with or without NIR irradiation by FCM. **c** Expression of apoptosis-related proteins in CT26 cells treated with PBS (control), RV, TiS_2_/RV, IR780-TiS_2_/RV, and IR780-TiS_2_/RV + NIR. Cytochrome c, cleaved caspase-3, and cleaved caspase-9 were tested by Western blot. **P < 0.01, compared with IR780-TiS_2_/RV, TiS_2_/RV + NIR, and IR780-TiS_2_ groups

To illustrate the underlying mechanism of NIR-triggered chemotherapy of IR780-TiS_2_/RV, we analyzed the cell death type, mitochondrial membrane potential (Ψm), and expression levels of key apoptosis-related proteins. Firstly, FCM was used to detect the cell death type using Annexin V-FITC/PI staining (Fig. 5a). At the same RV concentration, free RV, TiS_2_/RV, IR780-TiS_2_/RV, and IR780-TiS_2_/RV + NIR could all induce apoptosis, mainly due to the presence of RV. Indeed, RV has been reported to have the ability to induce apoptosis. Of these treatments, IR780-TiS_2_/RV + NIR showed the highest apoptosis rate of 90.8%. Apoptosis signaling pathways were investigated next. A decrease of Ψm has been reported as the key event in the mitochondrial (intrinsic) apoptotic pathway [40]. At the same RV concentration, IR780-TiS_2_/RV + NIR decreased Ψm by about 85%, which was more significant than a Ψm decrease induced by IR780-TiS_2_, TiS_2_/RV with or without NIR, and free RV (Fig. 5b). This experiment indicates that IR780-TiS_2_/RV + NIR induced cell death which was mediated via the mitochondrial (intrinsic) apoptotic pathway [41]. Next, the expression of proteins critical for apoptosis, specifically cytochrome c (cyto c), caspase 9, and caspase 3, was detected via a Western blot assay. The cells treated with IR780-TiS_2_/RV + NIR expressed more cytosolic cyto c than those treated with RV, IR780-TiS_2_/RV, or IR780-TiS_2_/RV (Fig. 5c). The translocation of cyto c is the most important initiator of the caspase cascade [42–44]. Consequently, the expression of cleaved caspase 9 and cleaved caspase 3 was considerably raised in the IR780-TiS_2_/RV + NIR-treated cells. Together, these data clearly suggest that apoptosis was mediated by the mitochondrial (intrinsic) pathway.

### **In Vivo** NIR-Triggered Tumor Chemotherapy

**Fig. 6:**
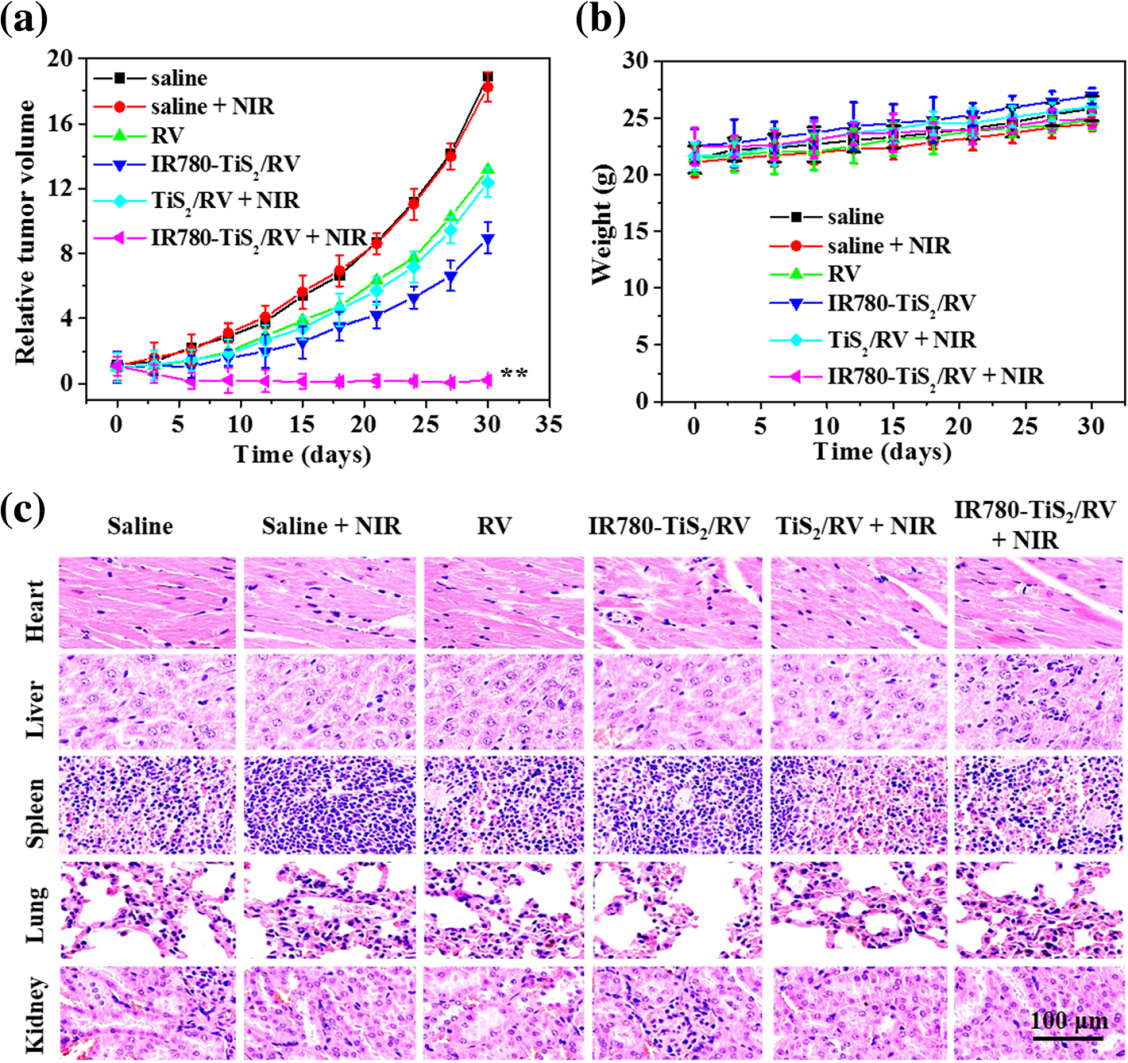
**a** The growth profile of CT26 xenografted tumors after intravenous injection of saline (control), RV, TiS_2_/RV, and IR780-TiS_2_/RV with or without a 3-min NIR irradiation (808 nm, 0.3 W/cm^2^). **b** Body weight of tumor-bearing mice after various treatments. **c** H&E images of major organs of all treated mice after 30 days of treatment. **P < 0.01, compared with saline, saline + NIR, RV, TiS_2_/RV + NIR, and IR780-TiS_2_/RV groups

To evaluate the efficacy of the NIR-triggered chemotherapy in vivo, CT26 tumor-bearing mice were treated with saline, saline + NIR, RV, IR780-TiS_2_/RV, TiS_2_/RV + NIR, and IR780-TiS_2_/RV + NIR. The tumor size in the IR780-TiS_2_/RV + NIR group significantly decreased, and the tumor almost disappeared after 30 days of treatment, while in the remaining groups, the tumor volume showed a rising trend (Fig. 6a). During the treatment, there had been no significant difference in body weight between the groups (Fig. 6b). Finally, H&E imaging revealed no noticeable tissue toxicity or abnormality in all tested groups (Fig. 6c). These results demonstrate that the IR780-TiS_2_/RV nanocomposite has an outstanding NIR-triggered anti-tumor efficacy and a low systemic toxicity. In addition, the biodistribution of the IR780-TiS_2_/RV was evaluated in vivo. As shown in Additional file 1: Figure S4, the nanoparticles were mostly entered into the liver system and maybe metabolized by the system [45].

## Conclusion

In summary, we have developed a mitochondria-targeted and RV-loaded nanoplatform based on TiS_2_ nanosheets for a NIR-triggered drug release and enhanced tumor chemotherapy. The as-prepared IR780-TiS_2_/RV with flake-like morphology showed good stability and biocompatibility. Owing to the mitochondria-targeted ability of IR780, IR780-TiS_2_/RV could selectively accumulate in tumor cell mitochondria and where it could release RV when triggered by the NIR irradiation. The released RV facilitated the mitochondrial membrane potential decrease, cyto c release, and, subsequently, initiated a cascade of caspase reactions to promote tumor cell apoptosis through the mitochondrial signaling pathway. In vitro and in vivo results demonstrated that IR780-TiS_2_/RV exhibited an efficacious NIR-triggered tumor chemotherapy without a significant tissue toxicity. These results suggest that IR780-TiS_2_/RV could be a promising chemotherapeutic agent in clinical practice.

## Additional File

Additional file 1: **Figure S1.** The low resolution TEM image of TiS_2_ nanosheets. **Figure S2.** XRD pattern of TiS_2_ nanosheets. **Figure S3.** The FTIR spectra of the TiS_2_ nanosheets, IR780-TiS_2_, and IR780-TiS_2_/RV, respectively. **Figure S4.** Ti content in tumor and major organs, including the heart, liver, spleen, lung, and kidney of IR780-TiS_2_/RV. (DOCX 260 kb)

## Data Availability

The conclusions made in this manuscript are based on the data which are all presented and shown in this paper.

## Abbreviations

IR780: IR-780 iodide
BET: Brunauer-Emmett-Teller
BSA: Bovine serum albumin
BSA: Bovine serum albumin
CCK-8: Cell Counting Kit-8
CLSM: Confocal laser scanning microscopy
cyto c: Cytochrome c
DMEM: Dulbecco’s modified Eagle’s medium
DMSO: Dimethylsulfoxide
FBS: Fetal bovine serum
FCM: Flow cytometry
FITC: Fluorescein isothiocyanate
FTIR: Fourier Transform Infrared Spectroscopy
NIR: Near-infrared
PBS: Phosphate-buffered saline
RV: Resveratrol
TEA: Triethylamine
TiS_2_: Titanium disulfide
