# Supplementary material for: Mitochondria-Targeted and Resveratrol-Loaded Dual-Function Titanium Disulfide Nanosheets for Photothermal-Triggered Tumor Chemotherapy
Source: Nanoscale Res Lett. 2019 Jun 21;14:211. doi: 10.1186/s11671-019-3044-5 (PMC6588667; doi:10.1186/s11671-019-3044-5)
Supplement: Supplementary file 1 — Figure S1. The low resolution TEM image of TiS2 nanosheets. Figure S2. XRD pattern of TiS2 nanosheets. Figure S3. The FTIR spectra of the TiS2 nanosheets, IR780-TiS2, and IR780-TiS2/RV, respectively. Figure S4. Ti content in tumor and major organs, including the heart, liver, spleen, lung, and kidney of IR780-TiS2/RV. (DOCX 260 kb) [file 11671_2019_3044_MOESM1_ESM.docx]

**Supplementary materials**

**Mitochondria-targeted and resveratrol-loaded dual-function titanium disulfide nanosheets for photothermal-triggered tumor chemotherapy**

Sen Xiang^1^*, Kaifang Zhang^1^, Guanghua Yang^1^, Dongdong Gao^1^, Chen Zeng^1^, Miao He^1^

^1^ The first department of oncology, Zhumadian central hospital, Zhumadian 463000, China

Correspondence: Sen Xiang

Email: [doc_xsen@163.com](mailto:doc_xsen@163.com)

Add: 747 zhumadian zhonghua road, Zhumadian 463000, China

Tel: +86 13839928053


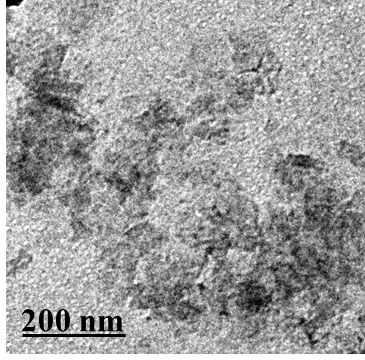


Figure S1. The low resolution TEM image of TiS_2_ nanosheets.


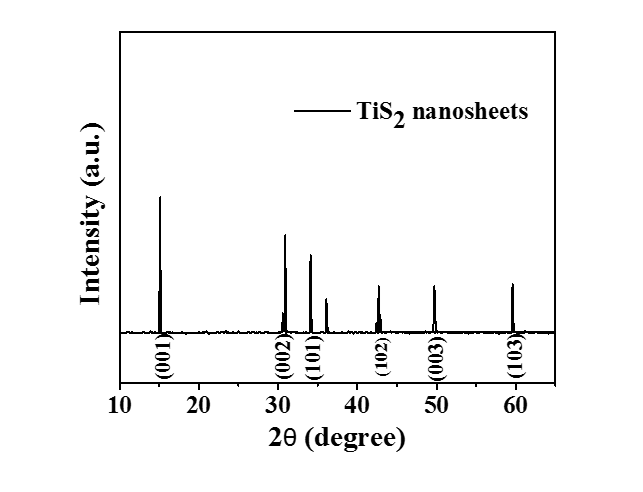


Figure S2. XRD pattern of TiS_2_ nanosheets.


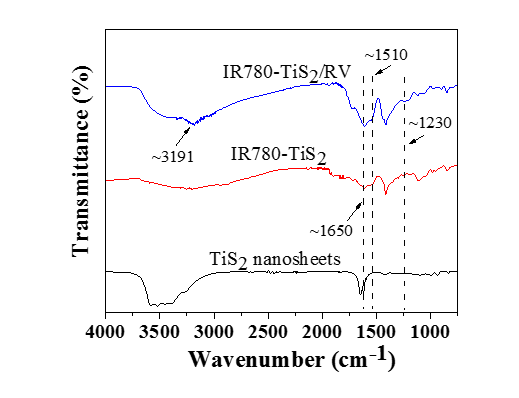


Figure S3. The FTIR spectra of the TiS_2_ nanosheets, IR780-TiS_2_, and IR780-TiS_2_/RV, respectively.


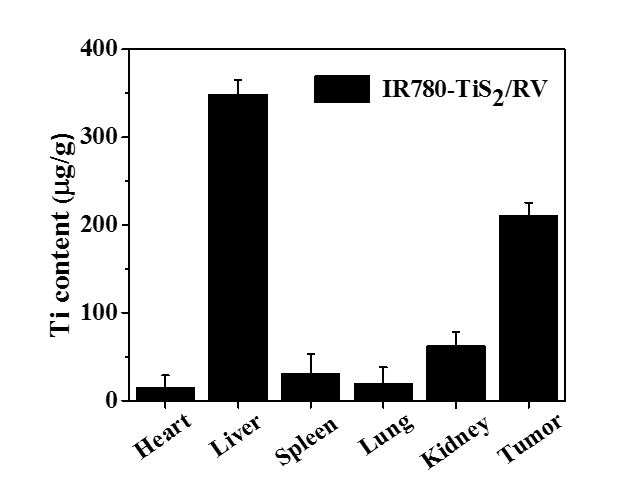


Figure S4. Ti content in tumor and major organs, including heart, liver, spleen, lung, and kidney of IR780-TiS_2_/RV.
